# Partitioning the efficiency of utilization of amino acids in growing broilers: Multiple linear regression and multivariate approaches

**DOI:** 10.1371/journal.pone.0208488

**Published:** 2018-12-12

**Authors:** Matheus De Paula Reis, Nilva Kazue Sakomura, Izabelle A. M. A. Teixeira, Edney Pereira da Silva, Ermias Kebreab

**Affiliations:** 1 Universidade Estadual Paulista (Unesp), Faculdade de Ciências Agrárias e Veterinárias, Jaboticabal, São Paulo, Brazil; 2 Department of Animal Science, University of California, Davis, California, United States of America; Tokat Gaziosmanpasa University, TURKEY

## Abstract

Determining the efficiency of amino acid (AA) utilization in growing animals is crucial to estimate their requirement accurately. In broiler chickens, the composition of AA in feather is different from feather-free body and the proportion of feathers will change along broiler’s growth, which may impact the efficiency of utilization on AA consumed. Therefore, in order to establish a method that predicts the efficiency of utilization for feather-free body and feather, two approaches were evaluated: a multiple linear regression and a multivariate analysis. Additionally, a new factorial model was proposed to predict AA requirements in broiler chickens. Data from 13 trials that evaluated the requirements for lysine (Lys), sulphur AA (SAA), threonine (Thr), and valine (Val) in male broilers were used for the analyses. Both methods of analysis were consistent in showing that the efficiency of utilization in feather-free body and feather were different. Using multiple linear regression, the values of efficiency of utilization estimated in feather-free body were 0.68, 0.72, 0.81, 0.79 (mg of amino acid deposited / mg of amino acid consumed above maintenance) and in feather were 0.58, 0.77, 0.78, and 1.57 (mg/mg) for Lys, SAA, Thr, and Val, respectively. Applying the multivariate approach, the corresponding predicted values were 0.68, 0.67, 4.23, 0.27 (mg/mg) in feather-free body and 1.16, 0.86, 0.16, and 1.10 (mg/mg) in feather, respectively. According to the results, efficiency of utilization may be related, to some extent, on the concentration determined in each tissue. The uncertainty around the amount of AA consumed for gain directed to feather-free body or feather deposition could be a limitation for multivariate analyses. The results indicated that multiple linear regression predictions may be better estimates of utilization efficiency. However, more studies are needed to elucidate the effect of age on deposition and partitioning of dietary AA in different parts of the broiler.

## Introduction

Nutrient metabolism in livestock is complex due to several interacting factors (e.g. rate of growth, environment, genotype, food composition, feed intake, etc). It is crucial to understand the complexity because this will lead to better matching of animal requirement to nutrient supply and consequently, reduced nutrient excretion to the environment [1, 2]. In this context, appropriate mathematical modeling techniques applied to animal production helps predict how the nutrient supplied to the animal would be utilized for various functions [3]. Factorial models that separate nutrient requirement for maintenance and tissue deposition have been used to study how nutrients are utilized and deposited in different parts of the animal. An overview of factorial models applied to poultry production is given by Sakomura and colleagues [4].

Protein and energy are expensive components of a poultry diet. Moreover, the amount and form of crude protein or synthetic amino acid supply in feed could make the difference between economic gain or loss to producers. In addition, the digestibility of protein or synthetic amino acid source and the amount offered to the animal will have implications for nitrogen excretion to the environment [2]. Evaluation of amino acid (AA) requirements involves knowledge of efficiency of utilization, which could be determined through nitrogen balance technique, where the AA deposition is regressed against consumption [4]. In this sense, the intake of amino acid (i.e. AA requirement) could be determined based on factorial models that considers the AA deposition and the AA required for maintenance, which indicate a straight influence of amino acid utilization over it requirement. In broilers, the AA deposition could be divided in feather-free body and feathers [4], suggesting an evaluation of the efficiency of utilization separately for each one of those parts. In addition, amino acid composition in feather-free body and feathers of broiler chickens varies considerably [5–7], therefore, separate determination of efficiency of utilization may increase the accuracy of the models developed to predict AA requirement. Multiple linear regression [8] and multivariate analyses [9] are statistical techniques traditionally used to determine the efficiency of energy deposited as protein and lipid [10–12]. This study aims to evaluate both techniques in their ability to describe AA partitioning.

We hypothesize that adjusting the factorial models described by Sakomura and colleagues [4] to account for an efficiency of utilization for feather-free body and feather, independently, allows for improved estimation of AA requirements in broiler chickens. The aim of the study was to determine the efficiency of utilization of Lysine (Lys), Sulphur Amino Acids (SAA), Threonine (Thr), and Valine (Val) for feather-free body and feathers in broiler chickens using a multiple linear regression and a multivariate approach. Furthermore, we also aim to develop adjusted factorial models to account for distinct efficiencies in feather-free body and feathers.

## Material and methods

The data used in this analysis were obtained from two studies conducted at the Poultry Science Laboratory of Faculdade de Ciências Agrárias e Veterinárias, UNESP–Univ Estadual Paulista, Jaboticabal Campus in Sao Paulo, Brazil [2,13] and one study conducted at Faculty of Veterinary Medicine and Animal Science, University of São Paulo, Campus Pirassununga [14]. All studies were approved by the Ethical Committee on the Use of Animals of the UNESP–Univ Estadual Paulista and adhere to its principles. All the procedures and facilities used to evaluate the AA were similar and have been fully described in previous studies [2, 13, 14], so a brief description follows.

### Database

A database from 13 dose response trials, using male Cobb500 broiler chickens was used to determine the efficiency of utilization for Lys, SAA, Thr, and Val. Each trial was designed to represent a specific stage in broiler chickens’ growth, however, to simplify the demonstration of the technique, rather than determine the efficiency of utilization by age, only one value was estimated for each AA, considering all ages at the same time. The Lys study [13] consisted of 4 dose response trials, each with 5 levels of digestible Lys. The levels studied ranged from 0.98 to 1.40% (1 to 8 days), 0.84 to 1.21% (8 to 21 days), 0.79 to 1.13% (21 to 35 days), and 0.75 to 1.07% (35 to 42 days), for growing broilers. Data from [2] were used to evaluate the efficiency of utilization using seven levels in three trials ranging from 0.25 to 0.90% (1 to 14 days), 0.23 to 0.81% (14 to 28 days), and 0.21 to 0.75% (28 to 42 days) of digestible SAA and 0.15 to 1.0% (1 to 14 days), 0.13 to 0.89% (14 to 28 days), and 0.12 to 0.82% (28 to 42 days) of digestible Thr. Similarly, data from [14] were used to determine the efficiency of utilization of Val in three trials, with seven levels of Val ranging from 0.46 to 1.15% (1 to 14 days), 0.40 to 1.03% (14 to 28 days), and 0.38 to 0.95% (28 to 42 days) of digestible Val. All experimental diets were produced using dilution technique [15], which consisted of formulating two diets, a diet with high content of nitrogen, and nitrogen-free diet. All diets were analyzed for gross energy and AA content [2, 13, 14].

The response variables used to perform all analyses were the AA intake (*Iaa*) and the amount of AA deposited in feather-free body and feather. In order to determine the deposition of the AA, a comparative slaughter technique was applied. All trials conducted to evaluate the amino acids were designed and developed by Sakomura and colleagues [2, 13, 14], following procedures described in previous studies [16].

The data used to perform statistical analyses were based on pens of 20 birds. Two birds per pen were randomly sampled to quantify the amino acid deposition. For Lys experiment, each level was replicated 6 times, in the SAA and Thr trials each level was replicated 4 times, and in the Val studies, each level was replicated 7 times. Outliers and normality assumptions were assessed using the Proc Univariate and Proc Means procedures of SAS (SAS Institute Inc., Cary, NC). To determine AA intake for gain (*Iaa*_*g*, *mg/bird/d*_), the AA consumed above maintenance was calculated as follows:

$${Iaa}_{g}=Iaa-{Iaa}_{m}$$ [Eq 1]

where *Iaa*_*m*_ is the intake of AA for maintenance.

The *Iaa*_*m*_ for each AA evaluated was determined by nitrogen balance technique in previous studies expressed as mg per protein unit (*BP*_*m*_^0.73^ kg x *u*), where *BP*_*m*_ is body protein at maturity and *u* is the degree of maturity of the bird (*u* = *BP*/*BP*_*m*_). The values determined were: 151 mg/*BP*_*m*_^0.73^ kg/day [17], 87.2 mg/*BP*_*m*_^0.73^ kg /day [18], 75.5 mg/*BP*_*m*_^0.73^ kg /day [18], and 247 mg/*BP*_*m*_^0.73^ kg /day [19], for the AA Lys, SAA, Thr, and Val, respectively. In addition, AA contents in feather-free body and feather were determined using high‐performance liquid chromatography (HPLC), obtaining values of 7.6, 3.6, 4.2, and 4.4 mg/100 g of protein in feather-free body and 1.8, 7.6, 4.2, and 6.0 mg/100 g of protein in feathers, for Lys, SAA, Thr, and Val, respectively.

The experiments in the database aimed to determine the amount of AA intake necessary for maximum deposition, therefore, all trials in this study had 5 (for Lys) or 7 levels of digestible AA, in which at least the last level was above the maxima, reaching a plateau where the slope is zero, which is no true for the efficiency of utilization. Thus, levels that were part of the plateau were identified and then removed. In this context, data were analyzed using a broken line method adjusting the *Iaa*_*g*_ in function of AA deposition, according to the following equation:

$${AA}_{d}=L+U*(R-X)$$ [Eq 2]

where *AA*_*d*_ is the AA deposition in feather-free body or in feather; *L* is the maximum AA deposition obtained with incremental levels of AA intake; *U* is the slope of the linear segment before plateau; *R* is the amount of AA intake necessary to reach the maximum AA deposition; *X* is the level of AA intake. Eq 2 is defined by a condition where: if *X* is less than *R*, the equation assumes a first-degree linear regression behavior. If the value of *X* is equal or higher than *R*, (*R* − *X*) is considered to be zero, consequently, the AA deposited is equal to *L*. This procedure was applied for both feather and feather-free body, and the levels used were always defined by the equation that gives the lower value of *L*. For example, if the amount of AA necessary to maximize deposition in feather-free body was within level 5 and for feather was within level 4, only levels from 1 to 4 were used to determine the efficiency of utilization.

### Multiple linear regression analyses

Data from each AA study was organized together to determine the efficiency of utilization. A multiple linear regression [8, 11], without intercept was fitted according to Eq 3:

$${Iaa}_{g}=b1*Bff+b2*Feather$$ [Eq 3]

where *b1* and *b2* are the amount of *Iaa*_*g*_ (mg/bird/day) deposited in feather-free body (*Bff*) and feather, respectively. The values *b1* and *b2* could be interpreted as the AA conversion ratio. Thus, the reciprocal of this value is the efficiency of utilization:

$${EU}_{Bff}=\frac{1}{b1}$$ [Eq 4]

$${EU}_{Feather}=\frac{1}{b2}$$ [Eq 5]

where, *EU*_*Bff*_ and *EU*_*Feather*_ are the efficiency of utilization (mg of amino acid deposited per mg of amino acid consumed above maintenance) of the AA for feather-free body and feather deposition_,_ respectively.

### Multivariate regression analyses

Assuming that *Iaa*_*g*_ goes to deposition in feather-free body and feather, the data were used to fit two equations:

$${Bff}_{d}=Z*k_{Bff}*{Iaa}_{g}$$ [Eq 6]

$$F_{d}=(1-Z)*k_{f}*{Iaa}_{g}$$ [Eq 7]

where the *Bff*_*d*_ and *F*_*d*_ are the AA deposited in feather-free body and feather, respectively. The parameter *Z* represents the fraction of the AA consumed above maintenance that is used for *Bff*_*d*_, likewise, the remaining fraction (1 − *Z*) represent the AA consumed above maintenance used for feather deposition, considering their respective efficiencies of utilization (*K*_*Bff*_ and *K*_*f*_ in mg of amino acid deposited per mg of amino acid consumed above maintenance, respectively).

Broilers have different proportion of feather-free body and feather, which is related to some degree to age. In addition, there is an influence of the AA composition in feather protein, which changes over time [5–7]. For those reasons, the parameter *Z* was assumed to have a linear relationship with age:

Z=a+b*age [Eq 8]

### Factorial model

The main objective of this work was to develop a methodology to estimate AA efficiency of utilization for feather-free body and feathers, given the difference in AA composition between these parts [20]. Furthermore, the efficiency of utilization could be used in factorial models to improve estimates of AA requirements. In extant factorial models [21], in which the requirement of the AA for maintenance and for growth are separated into feather-free body and feather, the efficiencies of AA utilization for feather-free body and feathers could be also given separately:

$$AAi=\left[({AA}_{m}*{BP}_{m}^{0.73}*u)+(FL*FP*{AA}_{f})\right]+[\frac{{(AA}_{b}*BPD)}{k_{Bff}}+\frac{{(AA}_{f}*FPD)}{k_{f}}]$$ [Eq 9]

where *AA*_*m*_ is the requirement of the AA for maintenance expressed as body protein (*BP*) ($\mathrm{mg}/{BP}_{m}^{0.73}$), *FL* refers to feather loss and it is equivalent to 0.001 g/g feather per day [20], *FP* is the feather protein weight (g), *AA*_*f*_ is the AA content in the protein fraction found in feather, *AA*_*b*_ is the AA content in the protein fraction of feather-free body (mg/g), *BPD* is the rate of protein deposition in the feather-free body (g/day), *K*_*Bff*_ is the efficiency of AA utilization (mg/mg) for deposition in feather-free body, *FPD* is the rate of protein deposition in the feather, and *K*_*f*_ is the efficiency of AA utilization (mg/mg) for deposition in feather. The parameters of the factorial models were determined in the studies previously mentioned. Eq 9 has four components: the first part represents the requirement for maintenance for feather free body $\left({AA}_{m}*{BP}_{m}^{0.73}*u\right)$, with parameter values determined in previous studies [17–19]. The maintenance for feathers is represented by feather loss (*FL* * *FP* * *AA*_*f*_). The amount of AA required for growth of the feather-free body is represented by ($\frac{{(AA}_{b}*BPD)}{k_{Bff}}$). Likewise, the AA required for feather growth is represented by ($\frac{{(AA}_{f}*FPD)}{k_{f}}$). Using the parameters and maintenance values determined in our previous studies (Table 1), the model was tested for each AA using the efficiency of utilization for feather-free body and feather estimated by multiple linear regression and multivariate techniques.

**Table 1:** Mature weight (Wm), deposition ratio (B), and initial weight (Wi) determined for body protein (BP) and feather protein (FP) in broiler chickens.

Parameters*	Wm (kg)	B (day)	Wi (kg)
BP	1.300	0.049	0.004
FP	0.353	0.045	0.0015

*Parameters determined in previous studies [17–19].

### Statistical analysis

All statistical procedures were conducted using SAS version 9.3 (SAS Institute Inc., Cary, NC). One-way ANOVA analysis using a pen as an experimental unit was conducted in a completely randomized design according to Proc GLM procedure. The NLIN and REG procedures were applied to fit broken line [22] and multiple linear regression, respectively. The multivariate analysis was performed using NLIN procedure [10]. A weighted regression procedure was used to reduce the residual variances for *Bff*_*d*_ and *F*_*d*_ [10]. The Akaike information criteria (AIC) was applied as a good fit criterion as follows:

$$AIC=N*LOG\left(\frac{{SS}_{res}}{N}\right)+2*P$$ [Eq 10]

Where N is the total sample size, SS_Res_ the residual sum of squares and P the number of parameters involved in the model. The efficiencies were used in the factorial models reparametrized by [4].

## Results and discussion

The requirement of an AA in growing animals is given firstly by the necessity of the body to fulfill the AA turnover or the maintenance requirement. Since animal’s metabolism will not synthesize some AA or the synthesis will not be sufficient to meet the requirement for maintenance, there is a necessity to supply the animal with such AA, called essentials. Determination of AA required for maintenance and its utilization has been attempted by various researchers, however, the results are so far inconclusive [23], denoting a necessity of more studies in this area. In this study, the maintenance was expressed in protein unit, as it corrected for growing animals, because it accounts for the maturity degree of protein (*u*), which is the relation of protein weight at a specific age by the mature protein weight. Furthermore, AA are not required for lipid reserves and considering that the amount of lipid might change among individuals [24] it is reasonable to choose body protein as a scale for maintenance calculation.

**Fig 1:**
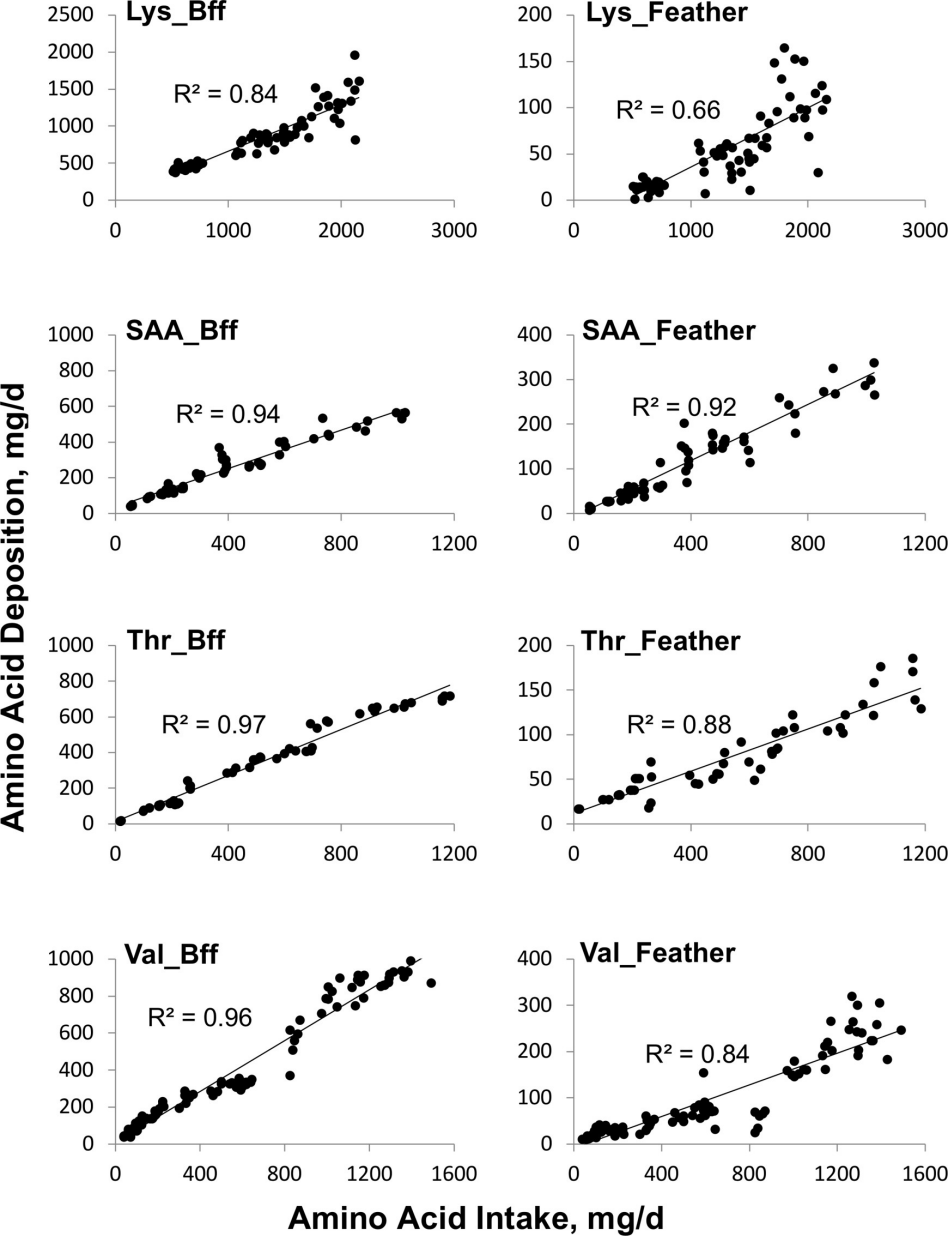
Adjustment of feather-free body (*Bff*) or feather amino acid deposition (mg/bird/day) in function of amino acid intake for gain (mg/bird/day).

For each AA, the average body protein weight measured was used to determine the *Iaa*_*m*_ using Eq 1, which allows determining *Iaa*_*g*_. The plots of AA deposition vs AA intake above maintenance (Fig 1) demonstrate that they are positively and linearly related.

The AA utilization could change according to the level in the diet [25–27], and low values of efficiency of utilization are observed when the intake of AA reach or exceed broiler requirement [27]. Based on this, Eq 2 was applied in order to determine the levels of AA intake that was below broilers’ requirement. In our previous studies [2, 13, 14], the efficiency how the amino acid is utilized in body deposition is determined plotting the amino acid deposition (Y axis) against amino acid intake (X axis), where the slope of the linear equation is the efficiency of utilization. In this study, the *Iaa*_*g*_ was split into deposition in feather-free body and feather to determine efficiency of utilization by multiple linear regression and multivariate approaches. The techniques were compared as which one allows an estimation of AA requirement closely related to observed data. The AIC showed in Table 2 demonstrate a better fit for Multiple linear regression.

**Table 2:** Akaike information criteria (AIC) for multivariate regression and multiple linear regression models adjusted to different amino acids.

Amino acid	AIC	
	Multivariate Regression	Multiple Regression
Methionine	622	481
Lysine	944	925
Threonine	504	444
Valine	832	831

### Multiple linear regression

Kielanowski [8] was one of the first to use a multiple linear regression technique to determine, among other parameters, the efficiencies of energy utilization for various purposes. The author considered energy consumption as the dependent variable and deposition of protein and lipid as independent variables, using the regression coefficient to explain the ratio in which the consumed energy is converted to body protein and body lipid. Later, the same technique was adopted by other authors [9, 11]. Using a similar approach but applied for AA (Eq 3), the *Iaa*_*g*_ (dependent variable) was regressed against the AA deposited in feather-free body and feathers (independent variable) to determine the reciprocal of the regression coefficient (Eq 4 and Eq 5), i.e., the efficiency of utilization for feather-free body and feathers (Table 3).

**Table 3:** Multiple linear regression equations adjusted with the intake of the amino acid for gain (*Iaa*_*g*_) and the deposition of the amino acid in feather-free body (*Bff*) and feather (*F*). Standard errors are given in parenthesis.

Amino acid	Equation	R^2^	Efficiency	
			Bff (mg/mg)	Feather (mg/mg)
Lysine	Iaa_g_ = 1.469 (±0.064)*Bff +1.864 (±0.730)*F	0.97	0.68	0.58
Sulfur-amino acid	Iaa_g_ = 1.384 (±0.163)*Bff +1.302 (±0.271)*F	0.99	0.72	0.77
Threonine	Iaa_g_ = 1.239 (±0.078)*Bff + 1.275 (±0.380)*F	0.99	0.81	0.78
Valine	Iaa_g_ = 1.270 (±0.071)*Bff + 0.638 (±0.309)*F	0.98	0.79	1.57

P-values of regression coefficients (b1 and b2) for feather-free body and feather estimated for lysine (< .001 ; 0.0124), Sulfur-amino acid (< .001 ; < .001), Threonine (< .001 ; 0.0014), and Valine (< .001 ; 0.0414).

The results indicate that the AA were utilized with similar efficiency for deposition in feather-free body compared to feathers, except for Val. The difference observed in efficiency of utilization of Lys between feather-free body and feather were approximately 15%. In our previous study the efficiency of utilization estimated for Lys, considering feather-free body plus feather was 0.77 (mg/mg) [28]. In this study, the efficiency of Lys utilization for feather-free body (0.68 mg/mg) and feather (0.58 mg/mg) were different to that reported previously for the whole body. Considering that the database in both studies was the same, the difference observed in the efficiency of utilization was due to the techniques applied to estimate how broiler chickens utilize the AA during growth. Nevertheless, Han and Baker [29] estimated values of 0.67 and 0.69 (mg/mg) for Lys utilization for broiler chickens with slow and fast growing rate, which is similar to our study. Furthermore, Lys is an essential AA mostly found in feather-free body (~7.0%) than in feathers (~2.0%), which may be related to some degree to the efficiency of utilization found in both parts in this study.

Regarding SAA, the difference in efficiency of utilization between body parts were lower as birds show an efficiency to deposit SAA in feathers 6% greater compared to feather-free body. The results estimated for SAA for feather-free body (0.72 mg/mg) and feathers (0.77 mg/mg) agreed closely with previous study [2] for the whole body (0.78 mg/mg). The concentration of 3.6 and 7.6 g SAA/100 g protein in feather-free body and feather, respectively, reinforces our hypothesis that the efficiency of utilization in different tissues may be related to each tissue concentration.

The results observed for Thr demonstrated the smallest difference for efficiency of utilization among parts, as the difference was only 4%, with the higher efficiency observed to deposit Thr in feather-free body. Furthermore, the data also suggest that a small difference in Thr concentration in feather-free body (4.2%) and feathers (4.4%) reflecting the small difference in efficiency of Thr utilization in feather-free body (0.82 mg/mg) and feather (0.78 mg/mg).

The efficiency of utilization of Val in feather was estimated to be greater than 1 and in feather-free body was 0.79 (mg/mg), even when the concentration for both tissues were 6.0 and 4.4%, respectively. An efficiency of utilization higher than 1 is only possible if a synthesis of Val is happening in this tissue. Catabolism of Val is initiated by aminotransferase enzyme found in abundance in skeletal muscle. This reaction is reversible and will produce α-ketoacid. However, to our knowledge there is no literature reporting a synthesis of Val by chickens. Furthermore, the oxidation of Val ketoacid will occur mainly in liver and is regulated by other factors such as leucine and energy levels of feed [30], the latter is related to the glycogenic characteristic of Val. In this sense, the particularity of Val catabolism could be influencing the estimated values obtained in this study, and more investigation should be done to elucidate this issue.

### Multivariate regression analyses

Using this approach, the efficiency of utilization for each AA were estimated, as well as the parameters *a* and *b*, used to determine the fraction of the AA consumed above maintenance (*Z*) that is used for feather-free body deposition (Table 4).

**Table 4:** Parameter estimates for describing each amino acid deposition in growing broilers as a function of intake of amino acid above maintenance (mg/bird/day) and age of broiler chickens according to multivariate model. Standard errors are given in parenthesis.

Amino acid	Parameters^2^			
	a	b	*K*_*Bff*_ (mg/mg)	*K*_*f*_ (mg/mg)
Lysine	0.991 (±0.04)	-0.001 (±0.002)	0.676 (±0.048)	1.168 (±2.098)
SAA^1^	0.715 (±0.286)	-0.001 (±0.001)	0.668 (±0.290)	0.858 (±0.798)
Threonine	0.151 (±1.359)	0.001 (±0.002)	4.230 (±37.772)	0.159 (±0.262)
Valine	2.059 (±2.516)	0.019 (±0.025)	0.274 (±0.338)	-0.101 (±0.204)

^1^SAA–Sulfur amino acid.

^2^Parameter a is the amount of amino acid consumed above maintenance directed for deposition in feather-free body of chickens at 8 days old for lysine or at 14 days old for other amino acids according to equation *Z* = *a* + *b* * (*age* − 8) for lysine and *Z* = *a* + *b* * (*age* − 14) for sulfur amino acids, threonine, and valine; b is the change in amino acid intake directed for feather-free body deposition (*Z*) due to a change in age; *K*_*Bff*_ is the efficiency of feather-free body amino acid deposition; and *K*_*f*_ is the efficiency of feather amino acid deposition.

The parameters from Eqs 6, 7 and 8 (Table 4), show good agreement for Lys and SAA with multiple regression model. However, it is notable that for Thr and Val the parameter estimates were not in line with results from the multiple regression model. The estimated Lys parameters indicate that in broiler chickens at 8 days old, 99% of Lys consumed above maintenance was directed towards feather-free body deposition. The fraction of Lys consumed above maintenance increased towards deposition in feathers after day 8 at 0.31% daily. The efficiency of utilization estimated for Lys deposition in feather-free body and feather were approximately 0.68 and 1.17 (mg/mg), respectively. The value of *K*_*f*_ obtained with multivariate model was different compared with multiple linear regression technique (0.58 mg/mg). For SAA, the estimated parameters suggest that at age 14, broilers’ will direct approximately 71% of the AA consumed above maintenance for deposition in feather-free body. Moreover, the *Iaa*_*g*_ directed for feather deposition increased after day 14 by 0.14%, according to age. The estimated value for Thr efficiency of utilization and the standard deviation indicate a poor adjustment with the model. In addition, the Thr efficiency of utilization in feather could be considered low, as the estimated value was approximately 16%. For Val, the estimated parameters also indicated a poor fit to multivariate model. The parameter that estimated the fraction of *Iaa*_*g*_ directed towards feather-free body deposition at 14 days of age (a), the negative value for efficiency of utilization and the AIC (832) obtained indicated poor fit to the model.

Considering the results for efficiency of utilization determined by multivariate approach (Table 4), a higher AIC values were estimated for this technique (Table 1), which indicate that was not the best approach tested herein. The simultaneous equation used take into account the maintenance and also the AA intake that is directed to feather-free body and feathers. In this sense, we used a linear equation to describe the fate of the AA consumed above maintenance that was directed for feather-free body, and by difference the amount directed towards feather deposition is found. However, this may not be the best approach to describe the fate of AA intake for gain, because the protein composition in feathers might change through age [5, 6].

To verify the influence of the parameter *Z* in the multivariate approach analysis, the proportion of the AA deposited in feather-free body in relation to the *Iaa*_*g*_ (parameter a), obtained at day 14 on Thr and Val trials, were fixed. Therefore, the parameter *a* from Eq 8 was set as 0.80 for Thr and Val (Table 5).

**Table 5:** Parameter estimates for describing each amino acid deposition in growing broilers as a function of intake of amino acid above maintenance (mg/bird/day) and age of broiler chickens according to multivariate model. Standard errors are given in a parenthesis.

Amino acid	Parameter^1^			
	a	b	*K*_*Bff*_ (mg/mg)	*K*_*f*_ (mg/mg)
Threonine	0.80	0.001 (±0.001)	0.812 (±0.021)	0.732 (±0.084)
Valine		0.001 (±0.001)	0.838 (±0.025)	0.925 (±0.133)

^1^Parameter a is the amount of amino acid consumed above maintenance that is directed to be deposited in feather free body at 14 days according to equation *Z* = *a* + *b* * (*age* − 14) for threonine, and valine; b is the change in amino acid intake for feather-free body deposition (*Z*) due to a change in age; *K*_*Bff*_ is the efficiency of feather-free body amino acid deposition; and *K*_*f*_ is the efficiency of feather amino acid deposition.

After adjustment, it was notable that the parameters *K*_*Bff*_ and *K*_*f*_ were better predicted, and the standard errors considerably reduced. The estimations made for Thr suggest that the broiler utilize the *Z* more efficiently than it does for (1 − *Z*), in other words, considering Iaa_g_ the best utilization rely when Thr is deposited in feather-free body, 0.83 mg of amino acid deposited for each one mg of amino acid consumed above maintenance. On the other hand, the efficiency of utilization determined for Val shows the opposite behavior, in which Val was deposited more efficiently in feather (~0.92) than in feather-free body (~0.84).

### Validation of procedures used to determine *K*_*Bff*_ and *K*_*f*_

Based on these results, a validation procedure was performed, considering the efficiency of utilization determined with multiple linear regression. The factorial models described by Sakomura and colleagues [4] were employed in using one efficiency of utilization (whole body) as described previously [2, 14, 27], or two efficiencies of utilizations, feather-free body and feather, determined in this study (adjusted factorial model). The requirements predicted by the equations are shown in Table 6.

$$Lys=[\left(151.2*{BP}_{m}^{-0.27}*BPt)+(0.01*FPt*20\right)]+[\frac{\left(70*BPD\right)}{0.68}+\frac{\left(20*FPD\right)}{0.58}]$$

$$Met+Cys=[\left(87.2*{BP}_{m}^{-0.27}*BPt)+(0.01*FPt*76\right)]+[\frac{\left(36*BPD\right)}{0.72}+\frac{\left(76*FPD\right)}{0.77}]$$

$$Thr=[\left(75.5*{BP}_{m}^{-0.27}*BPt)+(0.01*FPt*44\right)]+[\frac{\left(42*BPD\right)}{81}+\frac{\left(44*FPD\right)}{78}]$$

$$Val=[\left(219*{BP}_{m}^{-0.27}*BPt)+(0.01*FPt*60\right)]+[\frac{\left(44*BPD\right)}{79}+\frac{\left(60*FPD\right)}{100}]$$

**Table 6:** Lysine (Lys), sulfur-amino acid (SAA), threonine (Thr), and valine (Val) requirement for broiler chickens (mg/bird/day), determined by factorial approach using distinct efficiencies of utilization (*EU*) for feather-free body and feather, or same value for both parts.

Amino acid (mg)	Factorial model^a^	Age (days)					
		1 to 7	8 to 14	15 to 28	21 to 28	29 to 35	36 to 42
Lys	Adjusted	233	582	1080	1601	2006	2213
SAA		180	448	824	1208	1498	1642
Thr		148	369	681	1004	1251	1376
Val		180	448	820	1195	1472	1602
Lys	Sakomura et. al. 2015	203	506	939	1394	1749	1932
SAA		171	426	784	1149	1424	1561
Thr		164	408	753	1110	1383	1520
Val		187	466	858	1262	1569	1723

^a^ Factorial model described by Sakomura et. al. [4] and adjusted for distinct efficiency of utilization for feather-free body and feather (Adjusted) or equal efficiency of utilization [4].

**Fig 2:**
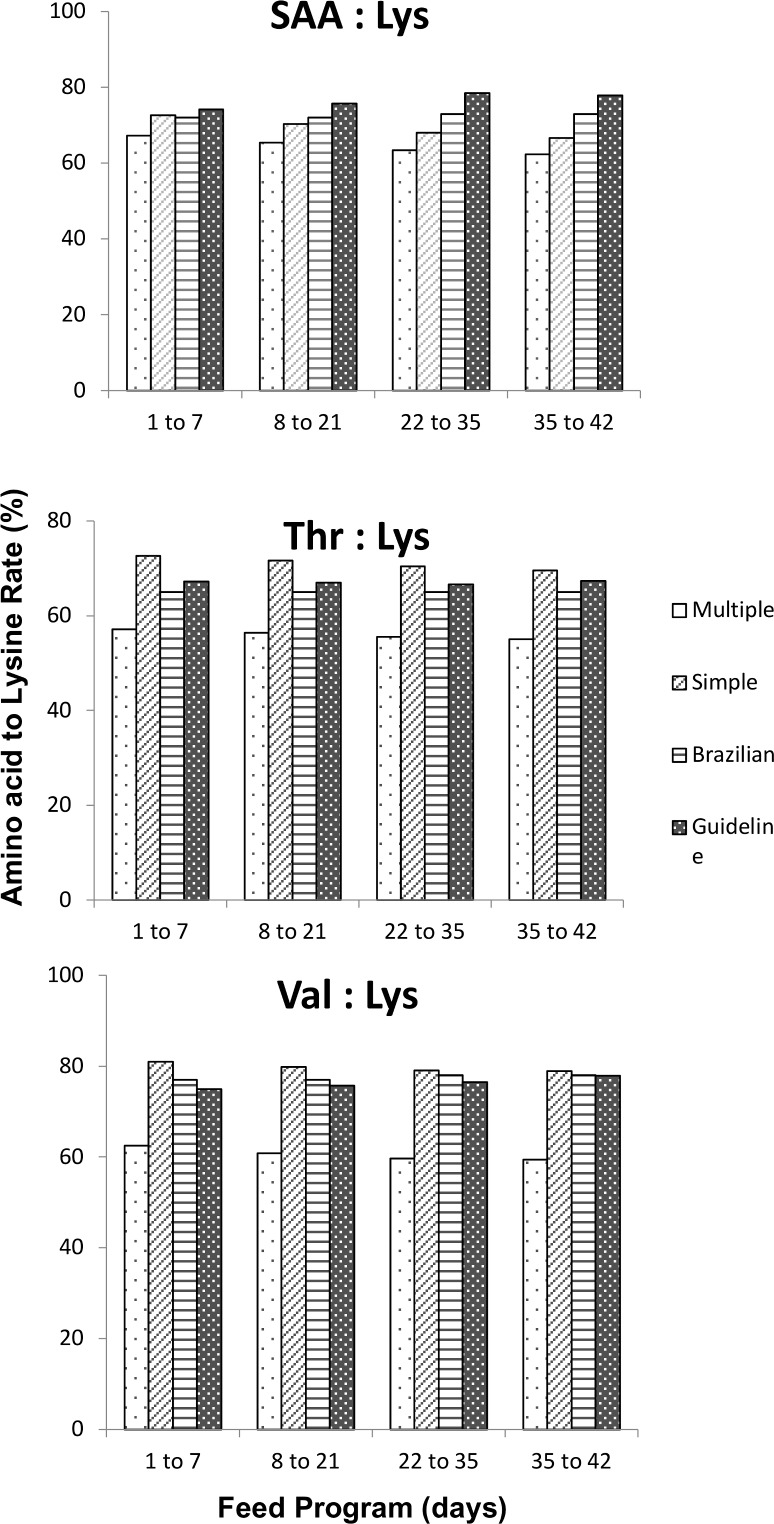
The ratio of sulfur-amino acid (SAA/Lys), threonine (Thr/Lys) and valine (Val/Lys) with lysine. The legend refers to adjusted and original factorial models, Brazilian Tables for Poultry and Swine (Brazilian) [31], and manual guide (Guideline) [32].

The requirement of AA determined when one or two efficiencies of utilization were used were similar, however, the values obtained when the multiple linear regression approach was used to predict the efficiency of utilization was slightly higher. The ratio of estimated AA requirement to Lys is demonstrated in Fig 2. The ratio was determined using the efficiencies obtained with original and adjusted factorial models, in other words, using one or two efficiencies of utilization to describe AA utilization. These findings were compared to Brazilian Tables for Poultry and Swine [31] and manual guide [32] (Fig 2).

In general, the ratio of AA with Lys predicted using the adjusted model was smaller than the values obtained when the original factorial model was used. On the other hand, the ratio obtained with adjusted factorial models was closely related to that published by Cobb-Vantress [32] and Rostagno et al. [31]. Advantages and disadvantages of both techniques were discussed by other researchers [11, 12, 33]. The simplicity of multiple linear regression may be the best advantage of this technique, on the other hand, some authors indicate that an intake of nutrient and its deposition on body tissues should be considered as independent and dependent variable, respectively. The opposite assumption is adopted when multiple linear regression is used to determine the efficiency of utilization of a nutrient in different parts of the animal’s body. To overcome this issue multivariate approach was developed and improved in the last decades [9, 10, 12, 33]. Instead of using one simple equation to describe energy deposition, the authors used two simultaneous equations, eliminating the need to declare dependent or independent variables. However, besides the success of multivariate technique in estimating efficiency of utilization for energy deposition as protein and fat, the same approach may not be the best way to determine AA deposition for poultry. One of the reasons for this issue could be the difficulty to understand the proportion of the nutrient consumed above maintenance and directed towards each tissue. In this study, we found that the complexity of this technique and uncertainty around some parameters determination may be the main disadvantages of the multivariate approach.

## Conclusion

According to the simulations performed using dose response available data, multiple linear regression was a better approach to determine the efficiency of utilization for Lys, SAA, Thr, and Val. The factorial model adjusted for two efficiencies of utilization predicted the requirement and the ratio of each AA with Lys well, suggesting that the efficiency of utilization for feather-free body and feather should be considered separately which may help nutritionists to predict the AA requirement more accurately.

## References

[1] Appuhamy JADRN, France J, Kebreab E. Models for predicting enteric methane emissions from dairy cows in North America, Europe, and Australia and New Zealand. Global Change Biology. 2016; 22: 3039–3056. 10.1111/gcb.13339 27148862 10.1111/gcb.13339

[2] Donato DCZ, Sakomura NK, Silva EP, Troni AR, Vargas L, Guagnoni MAN, et al Manipulation of dietary methionine+cysteine and threonine in broilers significantly decreases environmental nitrogen excretion. Animal. 2016; 10: 903–910. 10.1017/S175173111500289X 27076031 10.1017/S175173111500289X

[3] Gous RM. Modeling as a research tool in poultry science. Poultry Science. 2014; 93: 1–7. 10.3382/ps.2013-03466 24570415 10.3382/ps.2013-03466

[4] Sakomura NK, Silva EP, Dorigam JCP, Gous RM, St-Pierre N. Modeling amino acid requirements of poultry1. The Journal of Applied Poultry Research. 2015; 24: 267–282.

[5] Fisher ML, Leeson S, Morrison WD, Summers JD. Feather growth and feather composition of broiler chickens. Canadian Journal of Animal Science. 1981; 61: 769–773.

[6] Stilborn HL, Moran ET Jr., Gous RM, Harrison MD. Effect of Age on Feather Amino Acid Content in Two Broiler Strain Crosses and Sexes. The Journal of Applied Poultry Research. 1997 6: 205–209.

[7] Stilborn HL, Moran ET Jr., Gous RM, Harrison MD. Influence of age on carcass (feather-free) amino acid content for two broiler strain-crosses and sexes. The Journal of Applied Poultry Research. 2010; 19: 13–23.

[8] Kielanowski J. Estimates of the energy cost of protein deposition in growing animals. Energy metabolism. 1965 13: 121.

[9] Koong LJ. A New Method for Estimating Energetic Efficiencies. The Journal of Nutrition. 1977; 107: 1724–1728. 10.1093/jn/107.9.1724 894370 10.1093/jn/107.9.1724

[10] van Milgen J and Noblet J. Energy partitioning in growing pigs: the use of a multivariate model as an alternative for the factorial analysis. Journal of Animal Science. 1999; 77: 2154–2162. 10461994 10.2527/1999.7782154x

[11] Azevedo PA, van Milgen J, Leeson S, Bureau DP. Comparing efficiency of metabolizable energy utilization by rainbow trout (Oncorhynchus mykiss) and Atlantic salmon (Salmo salar) using factorial and multivariate approaches. Journal of Animal Science. 2005; 83: 842–851. 10.2527/2005.834842x 15753339 10.2527/2005.834842x

[12] Strathe AB, Danfær A, Chwalibog A, Sørensen H, Kebreab E. A multivariate nonlinear mixed effects method for analyzing energy partitioning in growing pigs. Journal of animal science. 2010; 88: 2361–2372. 10.2527/jas.2009-2065 20348377 10.2527/jas.2009-2065

[13] Siqueira JC, Sakomura NK, Gous RM, Teixeira IAMA, Fernandes JBK, Malheiros EB. Model to estimate lysine requirements of broilers Modelling nutrient digestion and utilisation in farm animals. p 306–314. Wageningen Academic Publishers 2011a.

[14] Ferreira NT, Albuquerque R, Sakomura NK, Dorigam JCP, Silva EP, Burbarelli MFC, et al The response of broilers during three periods of growth to dietary valine. Animal Feed Science and Technology. 2016; 214: 110–120.

[15] Fisher C and Morris TR. The determination of the methionine requirement of laying pullets by a diet dilution technique. British Poultry Science. 1970; 11: 1, 67–82. 10.1080/00071667008415793

[16] Sakomura NK and Rostagno HS. Métodos de pesquisa em nutrição de monogástricos. FUNEP, Jaboticabal. 2016.

[17] Siqueira JC, Sakomura NK, Rostagno HS, Bonato MA, Pinheiro SRF, Nascimento DCN. Exigência de lisina para mantença determinada com galos de diferentes genótipos. Revista Brasileira de Zootecnia. 2011b; 40: 812–820.

[18] Bonato M. A., Sakomura NK, Siqueira JC, Fernandes JBK, Gous RM. Maintenance requirements for methionine and cysteine, and threonine for poultry. South African Journal of Animal Science. 2011; 41: 209–222.

[19] de Lima M, Sakomura NK, Dorigam JCP, Silva EP, Ferreira NT, Fernandes JBK. Maintenance valine, isoleucine, and tryptophan requirements for poultry. Poultry science. 2016; 95: 842–850. 10.3382/ps/pev380 26769273 10.3382/ps/pev380

[20] Emmans GC. The growth of turkeys In: Recent Advances in Turkey Science, London, Butterworths 1989; p 135–166.

[21] Martin PA, Bradford GD, Gous RM. A formal method of determining the dietary amino acid requirements of laying‐type pullets during their growing period. British Poultry Science. 1994; 35: 709–724. 10.1080/00071669408417737 7719736 10.1080/00071669408417737

[22] Robbins KR, Saxton AM, Southern LL. Estimation of nutrient requirements using broken-line regression analysis. Journal of Animal Science. 2006; 84: E155–E165. 16582088 10.2527/2006.8413_supple155x

[23] Fisher C. An overview of poultry models. In: International Symposium: Modelling in Pig and Poultry Production, Jaboticabl, SP. 2013. p 1–25.

[24] Nonis M and Gous R. Broiler breeders utilise body lipid as an energy source. South African Journal of Animal Science. 2012; 41: 369–378.

[25] Heger J and Frydrych Z. Efficiency of utilization of essential amino acids in growing rats at different levels of intake. British Journal of Nutrition. 1985; 54: 499–508. 4063333 10.1079/bjn19850135

[26] Batterham ES, Andersen LM, Baigent DR, White E. Utilization of ileal digestible amino acids by growing pigs: Effect of dietary lysine concentration on efficiency of lysine retention. British Journal of Nutrition. 1990; 64: 81–94. 2119225 10.1079/bjn19900011

[27] Fatufe A, Timmler R, Rodehutscord M. Response to lysine intake in composition of body weight gain and efficiency of lysine utilization of growing male chickens from two genotypes. Poult Sci. 2004; 83: 1314–1324. 10.1093/ps/83.8.1314 15339006 10.1093/ps/83.8.1314

[28] Siqueira JC, Sakomura NK, Dourado LRB, Ezequiel JMB, Barbosa NAA, Fernandes JBK. Diet formulation techniques and lysine requirements of 1- to 22-day-old broilers. Revista Brasileira de Ciência Avícola. 2013; 15: 123–134.

[29] Han Y and Baker DH. Lysine Requirements of Fast- and Slow-Growing Broiler Chicks1. Poultry Science. 1991; 70: 2108–2114. 10.3382/ps.0702108 1956857 10.3382/ps.0702108

[30] D'Mello. Responses of growing poultry to amino acids In Amino acids in animal nutrition second edition (ed. D’Mello JPF), pp. 237–263. CABI Publishing, Wallingford, UK; 2003.

[31] Rostagno HS. Brazilian tables for poultry and swine Composition of Feedstuffs and Nutritional Requirements. 4rd ed. Brazil: UFV Viçosa: 488; 2017.

[32] Cobb-Vantress. Broiler performance and nutrition supplement. 2017.

[33] Pullar JD and Webster AJF. Heat loss and energy retention during growth in congenitally obese and lean rats. British Journal of Nutrition. 1974; 31: 377–392. 4835791 10.1079/bjn19740046

